# Intermittent Hypoxia Exposure Helps to Restore the Reduced Hemoglobin Concentration During Intense Exercise Training in Trained Swimmers

**DOI:** 10.3389/fphys.2021.736108

**Published:** 2021-11-29

**Authors:** Xiquan Weng, Jieru Lin, Yu Yuan, Baoxuan Lin, Weiwei Huang, Hiu Tung Tin, Jia Li, Xu Yan, Wentao Lin, Hao Chen

**Affiliations:** ^1^College of Exercise and Health, Guangzhou Sport University, Guangzhou, China; ^2^Tianjiu Research and Development Center for Exercise Nutrition and Foods, Hubei Key Laboratory of Sport Training and Monitoring, College of Health Science, Wuhan Sports University, Wuhan, China; ^3^College of Physical Education, Guangzhou Sport University, Guangzhou, China; ^4^Institute for Health and Sport, College of Health and Biomedicine, Victoria University, Melbourne, VIC, Australia; ^5^Australia Institute for Musculoskeletal Sciences, Melbourne, VIC, Australia

**Keywords:** hypoxia, IHE, erythropoietin, EPO, testosterone, hemoglobin

## Abstract

In prolonged intense exercise training, the training load of athletes may be reduced once their hemoglobin concentrations ([Hb]s) are decreased dramatically. We previously reported that intermittent hypoxia exposure (IHE) could be used to alleviate the decrease of [Hb] and help to maintain the training load in rats. To further explore the feasibility of applying IHE intervention to athletes during prolonged intense exercise training, 6 trained swimmers were recruited to conduct a 4-week IHE intervention at the intervals after their [Hb] dropped for 10% or more during their training season. IHE intervention lasted 1 h and took place once a day and five times a week. Hematological and hormonal parameters, including [Hb], red blood cells (RBC), hematocrit (Hct), reticulocytes, serum erythropoietin (EPO), testosterone (T) and cortisol (C) were examined. After the IHE intervention was launched, [Hb], RBC and Hct of the subjects were increased progressively with their maximum levels (*P* < 0.01) showing at the third or fourth week, respectively. An increase in reticulocyte count (*P* < 0.01) suggests that IHE intervention promotes erythropoiesis to increase [Hb]. Besides, serum level of EPO, the hormone known to stimulate erythropoiesis, was overall higher than that before the IHE intervention, although it was statistically insignificant. Furthermore, the serum level of T, another hormone known to stimulate erythropoiesis, was increased progressively with the maximum level showing at the fourth week. Collectively, this study further confirms that IHE intervention may be used as a new strategy to prevent intense exercise training-induced reductions in [Hb].

## Introduction

Blood hemoglobin (Hb) serves as a routinely used marker for monitoring intense exercise training and physical function (Gleeson, 2002; Halson et al., 2003). There is literature supporting that a decrease in hemoglobin concentration ([Hb]) can be caused by acute or prolonged plasma volume (PV) expansion which can aid the athletes adapt to exercise training (Kargotich et al., 1998). However, higher [Hb] usually leads to better performance (Calbet et al., 2006; Cai et al., 2019), as long as the hematocrit (Hct) is at the optimum range (Reinhart, 2016; Sitina et al., 2021). Actually, based on our long-term observation and other studies (Hasibeder et al., 1987; Zhao, 2003), the training load of athletes will usually be reduced once their [Hb] were 10% lower than their baselines. Seeking appropriate strategies to restore the decreased [Hb] could be able to prevent the reduction in training load during prolonged intense training periods (Weng et al., 2021).

Altitude training (hypoxic training) has been widely adopted by athletes to improve their aerobic capacity (Millet et al., 2010). Hypoxia can induce expression of genes regulated by hypoxia inducible factors (HIFs) and then stimulate erythropoietin (EPO) production, which ultimately promotes the synthesis of Hb (Haase, 2013). When laboratory rats were subjected to intense exercise training for 6 weeks, one hour of hypoxic exposure (simulated altitude: 3,000 m) at the interval of exercise from the fourth to the sixth week could prevent the 10% decrease in [Hb] (Weng et al., 2021). These results suggest that hypoxia exposure can be potentially used to slow or prevent intense exercise training-induced decrease of [Hb] in athletes. However, in the rat study, the physiological and biochemical parameters of the rats were not dynamically monitored. To further explore the feasibility of applying hypoxia exposure to athletes during intense exercise training period, 6 trained swimmers were recruited to conduct hypoxic exposure at the intervals during their intense exercise training period. The effects of hypoxic exposure on the levels of [Hb], RBC, hematocrit (Hct), reticulocytes, serum EPO, testosterone (T) and cortisol (C) in athletes undergoing intense exercise training were investigated.

## Materials and Methods

### Subjects

Male trained swimmers who were members of the Guangzhou Sport University Team with at least ten-year training experience participated in this study. This study was conducted during their training season. Since this study was based on the training monitoring of the Guangzhou Sport University Swimming Team during its training season lasting about half a year prior to the Guangdong Provincial University Games, the swimmers were not subjected to various training loads to induce low hemoglobin concentration in certain groups, as it may produce negative outcomes to their training. All swimmers were trained by their coaches, and conducted the same training program with the same relative intensity. Their [Hb]s were examined every Monday. If the instant [Hb] of a swimmer was 10% lower than his baseline [Hb], the athlete was subjected to the IHE intervention from the same day on. In total, 6 swimmers were subjected to the IHE intervention (mean ± SD: age, 20.85 ± 0.634 yr; body weight, 68.71 ± 2.22 kg; height, 182 ± 2.49 cm; training duration, 11.85 ± 0.96 yr). None of the subjects have hematologic diseases, liver diseases, kidney diseases, or endocrine disorders. None of the subjects smoked, drank alcohol, or were taking medication known to alter the hormonal response. The study was conducted according to the Declaration of Helsinki, and the study was approved by the Guangzhou Sport University Ethics Committee (ID number: 2018LCLL-11). All the subjects provided written informed consent to participate in this study.

### Training Routine

The swimmers performed 10 training sessions per week (usually two sessions on Monday, Tuesday, Thursday, and Friday, respectively, 1 session on Wednesday and Saturday, respectively, and a break on Sunday), ∼150 min per session. Each session included two parts: swimming training and strength training. In the swimming training, the swimmers were subjected to interval training, continuous training or sprint training, and they completed a mean value of 40.0 km swimming per week. About 50% of the training volume aimed for the aerobic power of the swimmers, 20% aimed for the anaerobic power, and the remaining 30% aimed for both aerobic and anaerobic power. The strength training part included dry-land training and in-water training. Dry-land training, which was conducted 2–3 times per week with 1 h each time, included squat, running, bench press, pull-up, etc. There were various forms of in-water training regimes, which were conducted twice per week with half an hour each time, such as tethered training with elastic bands.

### Intermittent Hypoxia Exposure Design

The IHE intervention sessions were conducted in the evening after daily training. The subjects seated comfortably and conducted IHE intervention by breathing through a face mask (hypoxia generator hyp-100, hypoxia company, New York, United States). A simulated altitude of 3,000 m was chosen based on our previous rat study (Weng et al., 2021). Each IHE intervention session lasted for one hour and the study lasted for 4 weeks (5 days/week). No special load or schedule changes were made specifically for the subjects before, during and after the study.

### Sample Collection and Measurement of Hematological Parameters

The baseline values for [Hb], red blood cell count (RBC) and hematocrit (Hct) were collected one week before the training season. On every Monday during the IHE intervention period, the venous blood samples of the subjects were collected with EDTA-containing or EDTA-lacking tubes. Immediately after collection, 1 ml whole blood was analyzed to measure hemotological parameters: [Hb], RBC, Hct and reticulocyte count using an automated cell counter (ADVIA120, Bayer AG, Germany). Another 4 ml blood samples was collected using EDTA-lacking tubes and centrifuged at 3,000 rpm/min for 10 min at room temperature to collect the serum for the measurement of EPO, testosterone (T) and cortisol (c) (see below).

### Measurement of Erythropoietin, Testosterone, and Cortisol

Serum EPO, T and C were measured using enzyme-linked immunosorbent assays (ELISA) according to the manufacturer’s guidelines (Catalog #: E01E0002, E11T0007 and E11C0008, respectively, Bluegene Biotech CO., LTD, Shanghai, China). Briefly, 100 μl of conjugate was added to each well in the plate, then 50 μl of standards, control, or sample were added to the plate and incubated for 1 h at 37°C. Each well was aspirated and rinsed with wash buffer for a total of five washes. Substrate (100 μl) was added to each well and incubated for 10–15 min at 37°C in dark. Stop solution (100 μl) was added to each well and the plate was read within 30 min. Plates were read at 450 nm on a VARIOSKAN FLASH (Thermo Fisher Scientific, MA, United States). Each sample was measured in duplicate.

### Statistics

Non-parametric analyses of the data were analyzed using the procedure of Friedman test to locate overall significant differences among various weeks. Significant differences between various weeks were then determined with the Wilcoxon test. All statistical calculations were performed using IBM SPSS Statistics for Windows (version 22). Data were expressed as mean ± standard deviation (SD). *P*-values less than 0.05 were considered statistically significant.

## Results

### The Hematological Parameters of the Subjects Were Increased During Intermittent Hypoxia Exposure Intervention

**FIGURE 1:**
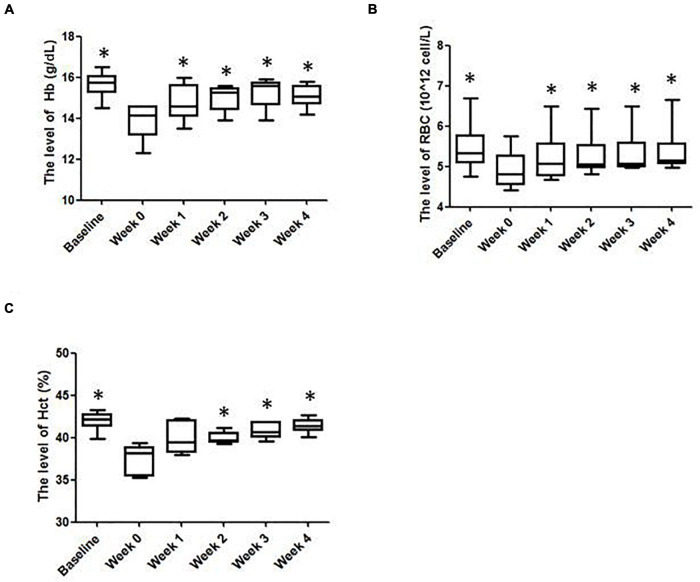
The hematological parameters of the subjects in various weeks. **(A)** Hemoglobin (Hb); **(B)** red blood cells (RBCs); **(C)** hematocrit (Hct) levels. ^∗^Significantly different from week 0, *P* < 0.05.

As shown in Figure 1A, after prolonged intense exercise training, the [Hb] of the subjects were about 11% lower than their baseline values. After the IHE intervention was launched, the [Hb] of the subjects increased progressively with the maximum level achieved at the third week. Similar outcomes also occurred for RBC and Hct. The RBC and Hct of the subjects increased progressively with both of their maximum levels achieved at the fourth week (Figures 1B,C).

Besides, the results of mean corpuscular volume (MCV), red cell distribution width (RDW), mean corpuscular hemoglobin concentration (MCHC), hemoglobin distribution width (HDW), mature red blood cell hemoglobin content (CH) and mean corpuscular hemoglobin (MCH) were slightly different from week to week and displayed no statistical significance (Table 1). Reticulocyte count can reflect the erythropoietic activity of the bone marrow (Cline and Berlin, 1963). Reticulocyte count can be reported as absolute reticulocyte count (Retic#) or as a reticulocyte percentage (i.e., reticulocytes per total RBCs examined, Retic%) (Riley et al., 2001). As shown in Table 2, after the IHE intervention was launched, the Retic# and Retic% of the subjects increased progressively. The Friedman test indicated that there was a statistically significant difference after the IHE intervention was launched for Retic# (*P* = 0.006), but not for Retic% (*P* = 0.054). As Retic# is an absolute value and more reliable, these results indicate that IHE intervention promoted erythropoiesis in the subjects.

**TABLE 1:** Some hematological parameters of subjects during IHE intervention.

**Group**	**MCV (fL)**	**RDW (%)**	**MCHC (g/dL)**	**HDW (g/dL)**	**CH (pg)**	**MCH (pg)**
Week 0	76.98 ± 7.83	12.78 ± 0.79	38.10 ± 1.49	2.66 ± 0.16	29.30 ± 3.85	28.53 ± 3.65
Week 1	77.10 ± 7.35	12.78 ± 0.67	37.98 ± 2.13	2.59 ± 0.09	29.28 ± 4.17	28.63 ± 3.91
Week 2	77.20 ± 7.94	12.85 ± 0.75	38.38 ± 1.38	2.65 ± 0.13	29.60 ± 3.91	28.85 ± 3.63
Week 3	78.13 ± 8.06	12.81 ± 0.73	38.08 ± 1.45	2.64 ± 0.15	29.68 ± 3.96	28.73 ± 3.72
Week 4	77.81 ± 8.01	12.78 ± 0.66	38.11 ± 1.45	2.63 ± 0.14	29.65 ± 3.93	28.65 ± 3.67

**TABLE 2:** The Retic# and Retic% of subjects during IHE intervention.

**Group**	**Retic#(10^9^/L)**	**Retic% (%)**
Week 0	50.23 ± 6.84	0.99 ± 0.15
Week 1	60.75 ± 15.09	1.20 ± 0.29
Week 2	66.13 ± 12.96^*^	1.28 ± 0.31
Week 3	63.53 ± 13.23^*^	1.25 ± 0.28
Week 4	65.81 ± 12.26^*^	1.27 ± 0.26

*Significantly different from week 0, *P* < 0.05.

### Erythropoietin and Testosterone of the Subjects Were Increased During Intermittent Hypoxia Exposure Intervention

**FIGURE 2:**
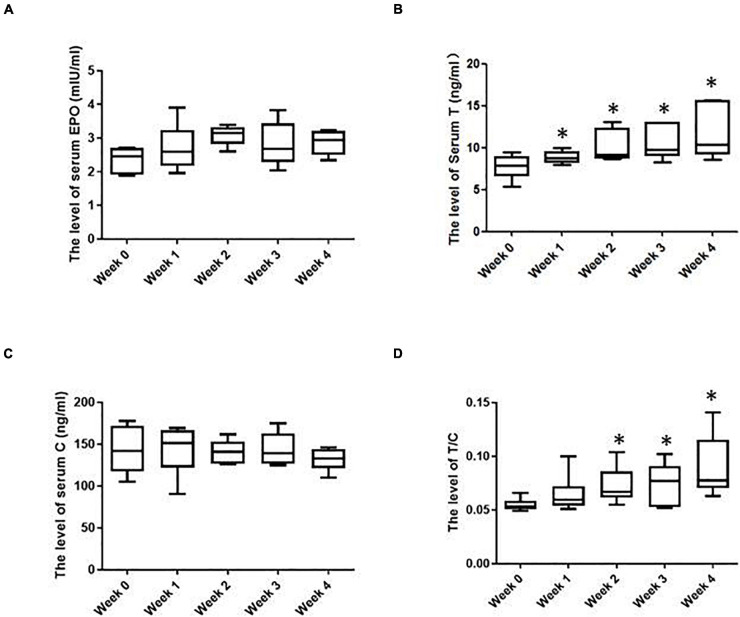
The hormonal parameters of the subjects in various weeks. **(A)** Serum erythropoietin (EPO) levels; **(B)** serum T levels; **(C)** serum C levels; **(D)** serum T/C levels. ^∗^Significantly different from week 0, *P* < 0.05.

As shown in Figure 2A, after IHE intervention was launched, the serum EPO level was overall higher than that before the IHE intervention, especially at the second week and the fourth week, although the Friedman test indicated that there was not a statistically significant difference for the EPO level. These results suggest that the IHE intervention might promote the production of serum EPO, which could subsequently stimulate erythropoiesis.

Besides EPO, testosterone (T) can also enhance erythropoiesis and [Hb] (Bachman et al., 2014; Cervi and Balitsky, 2017). As shown in Figure 2B, after IHE intervention was launched, the serum T of the subjects were increased progressively with the maximum level showing at the fourth week. T is known as an anabolic hormone and cortisol (C) is a catabolic hormone (Urhausen et al., 1995). On most occasions, their levels inside human bodies are well coordinated. Moreover, The T/C ratio can serve as an indicator of both the anabolic and catabolic balance and the fatigue state (Urhausen et al., 1995). The decrease of this ratio marks a potential physical function decrease (Urhausen et al., 1995). In this study, the level of the serum C remained overall stable before and during IHE intervention (Figure 2C). Therefore, the T/C ratio displayed the same shift as that of T (Figure 2D). These results suggest an increase of anabolic metabolism for the subjects during the HE intervention.

## Discussion

In our previous report, we adopted a moderate simulated altitude (3,000 m) and our data showed that it was sufficient to prevent low [Hb] in rats subjected to intense exercise training (Weng et al., 2021). This current study further confirmed the feasibility of this simulated altitude in athletes. In addition, we have previously found that one-hour IHE intervention was sufficient to prevent the appearance of low [Hb] in rats subjected to intense exercise training, although it may not be sufficient to prevent the decrease of RBC and Hct (Weng et al., 2021). However, in this study, one hour of normobaric hypoxia exposure can not only prevent the subjects from decreasing [Hb] during intense exercise training, but also blunt the decrease in RBC and Hct. These results suggest that humans might be more sensitive to the IHE intervention than rats.

There is literature in which roughtly 14 h per day are needed to stimulate erythropoiesis in healthy subjects in hypoxic interventions (Rusko et al., 2003; Brugniaux et al., 2006). However, in the current study, only one hour per day was needed. This design was justified as follows. Firstly, this study was conducted based on our research performed in laboratory rats previously published (Weng et al., 2021). In that study, rats undergoing intense exercise training were subjected to the IHE intervention lasting for three weeks for 1 h/day, 2 h/day and 3 h/day (the simulated height was 3,000 m), respectively. We found that IHE with different hours displayed comparable improvement in [Hb]. Therefore, we chose one-hour intervention in this study. Secondly, there is literature in which IHE intervention of 1–3 h is sufficient for enhanced erythropoiesis (Rodriguez et al., 1999, 2000; Casas et al., 2000). IHE intervention of different durations in various studies may be attributed to divergent physical characteristics of subjects and divergent types and/or intensities of exercise training involved in these studies (Millet et al., 2010; Viscor et al., 2018).

After IHE intervention was launched, the serum EPO level in the subjects reached the peak in the second week. However, the levels of RBC and Hb reached the peak in the fourth or third week, respectively, which indicated that the level shift of EPO was not strictly correlated with those of RBC and Hb. Moreover, the Friedman test failed to detect a statistically significant difference for the EPO level after IHE intervention was launched. These results showed that the increase in the serum EPO level upon hypoxic stimulation could be transient. These results are consistent with our previous study in rats, in which the serum EPO level did not display a significant increase compared with the exercise control group (*P* > 0.05) after the 3 weeks of IHE intervention, although [Hb] did increase (Weng et al., 2021). This can be attributed to the fact that it takes time from EPO secretion to the production of RBC and Hb. Therefore, the level shift of RBC, Hb and Hct occurred after that of EPO. The reason beyond this may be that the level increase of RBC and Hb can enhance the blood oxygen level, which imposes a negative feedback on the production of EPO (Maxwell et al., 1999; Ivan et al., 2001).

During the IHE intervention in this study, the level of serum T progressively increased with the maximum level showing at the fourth week. In previous studies, the level shifts of serum T during or upon hypoxia exposure displayed divergent results. For instance, the levels of serum T of 52 male soldiers decreased significantly after a 5,380 m altitude exposure for half a year, while increased significantly after another half a year (He et al., 2015). However, Ding et al., found that although 113 male subjects showed a significant increase in the serum T level one day after a 3,700 altitude exposure, their serum T levels fell below their baselines seven days later (Ding et al., 2018). These divergent results may be attributed to divergent physical characteristics of subjects and different duration of hypoxia exposure. It would be interesting to explore whether IHE intervention can keep increasing or maintaining the level of serum T after periods longer than 4 weeks in future studies.

This study has shown that the IHE intervention could partially restore the low [Hb] caused by intense exercise training in the subjects. We noticed that there is literature supporting that the [Hb] decrease during exercise training can be due to PV expansion (Kargotich et al., 1998) and that hypoxic expsure can reduce PV (Siebenmann et al., 2017). Thus, one potential interpretation of our results is that the IHE intervention may simply decrease PV and reverse the possible PV expansion induced by intense exercise training. However, there are studies reporting that the PV remains unchanged after IHE interventions. For instance, Rodriguez et al. (2000) reported an increase of [Hb] and Hct without signs of decreased PV (hemoconcentration). To support our statement, moreover, the “live high-train low” strategy has shown an increase of the hemoglobin mass without a decrease in PV (Wehrlin et al., 2006; Wehrlin and Marti, 2006; Saugy et al., 2014). On the other hand, we think that the recovery of [Hb] in the subjects benefited substantially from the stimulated erythropoiesis induced by the IHE intervention based on the following evidences. Firstly, the reticulocyte counts which can reflect the erythropoietic activity of the bone marrow (Cline and Berlin, 1963) of the subjects were increased significantly during the IHE intervention (Table 2); Secondly, the serum level of EPO, the hormone known to be induced by IHE to stimulate erythropoiesis, was overall higher than that before the IHE intervention, although it was statistically insignificant probably due to a relatively transient increase (Figure 2A); Finally, the serum level of T, another hormone known to be induced by IHE to stimulate erythropoiesis, was increased progressively during the IHE intervention (Figure 2B). Moreover, the T/C ratio, serving as an indicator of anabolic and catabolic balance and is of the fatigue state (Urhausen et al., 1995) displayed the same shift as that of T (Figure 2D), which may suggest, although indirectly, that the interaction between training-induced PV expansion and IHE-induced plasma loss can be overall beneficial for the subjects. Future investigation, including exercise tests, blood flow and blood volume measurements are warranted for clarification of outcomes of this interaction.

A few limitations of the current study have to be acknowledged. First of all, the sample size of 6 was not large and there was a lack of a control group. Due to the availability of swimmers with similar competition level and training history, we were not able to recruit more than 6 participants, nor a control group. Yet because of the strict selection criteria, we observed very similar physiological parameters among our participants, and similar response of the participants to the IHE intervention. Beside, this study was based on the training monitoring of the Guangzhou Sport University Swimming Team. Since this study was conducted during the training season before the Guangdong Provincial University Games, the subjects needed a uniform intervention for a fair competition, which made us unable to set a control group. Regardless, further studies with a big sample size and a control group are warranted. Secondly, the benefits of IHE need to be interpreted with caution. It is known that sport anemia is partially due to the increase of PV, which is associated improved microcirculation and better distribution of blood flow to exercising muscle (Fellmann, 1992). On the other hand, hypoxia is known to induce erythropoiesis, which can increase the number red blood cells and hematocrit (Haase, 2013), yet this increase could be transient and return to pre-hypoxia level in less than 2 weeks (Berglund, 1992). What we have demonstrated in the current study was that [Hb], red blood cell counts and hematocrit were decreased after intense training, while IHE was able to restore the reduced parameters partially or in full, but not above the pre-training baseline (Figure 1). Therefore, we could conclude that the IHE is not likely to increase the blood viscosity in these athletes. Moreover, we did not notice a decrease of training load or intensity for the swimmers, which could demonstrate the benefits of the IHE intervention. Finally, we did not monitor the [Hb]s of the subjects beyond the 4-week intervention period, nor study how long the restored [Hb] can maintain. Although the subjects can rejoin the IHE intervention whenever their [Hb]s drop again, future studies are warranted to explore the “shelf life” upon IHE intervention against the training-induced [Hb] decrease.

## Conclusion

One hour of normobaric hypoxia exposure (14.5% O2) each day during training intervals was sufficient to partially restore the low [Hb] in trained swimmers during prolonged exercise training. However, limitations, including a small sample size, lacking of a control, exercise tests and blood flow and blood volume measurements, are noticeable for this study. Future studies of more comprehensive design are warranted.

## Data Availability Statement

The raw data supporting the conclusions of this article will be made available by the authors, without undue reservation.

## Ethics Statement

The studies involving human participants were reviewed and approved by Guangzhou Sport University Ethics Committee. The participants provided their written informed consent to participate in this study.

## Author Contributions

XW and HC conceived and designed the research. XW, JeL, YY, BL, WH, WL, and HC conducted the experiments. XW, HTT, JaL, XY, WL, and HC analyzed the data. XW, XY, and HC prepared the original manuscript. XW, JeL, YY, HTT, JaL, XY, WL, and HC revised the manuscript. All authors read and approved the manuscript.

## Conflict of Interest

The authors declare that the research was conducted in the absence of any commercial or financial relationships that could be construed as a potential conflict of interest.

## Publisher’s Note

All claims expressed in this article are solely those of the authors and do not necessarily represent those of their affiliated organizations, or those of the publisher, the editors and the reviewers. Any product that may be evaluated in this article, or claim that may be made by its manufacturer, is not guaranteed or endorsed by the publisher.
